# In Memoriam: Jacques-Emile Dumont (1931–2023)

**DOI:** 10.1530/ETJ-23-0033

**Published:** 2023-03-17


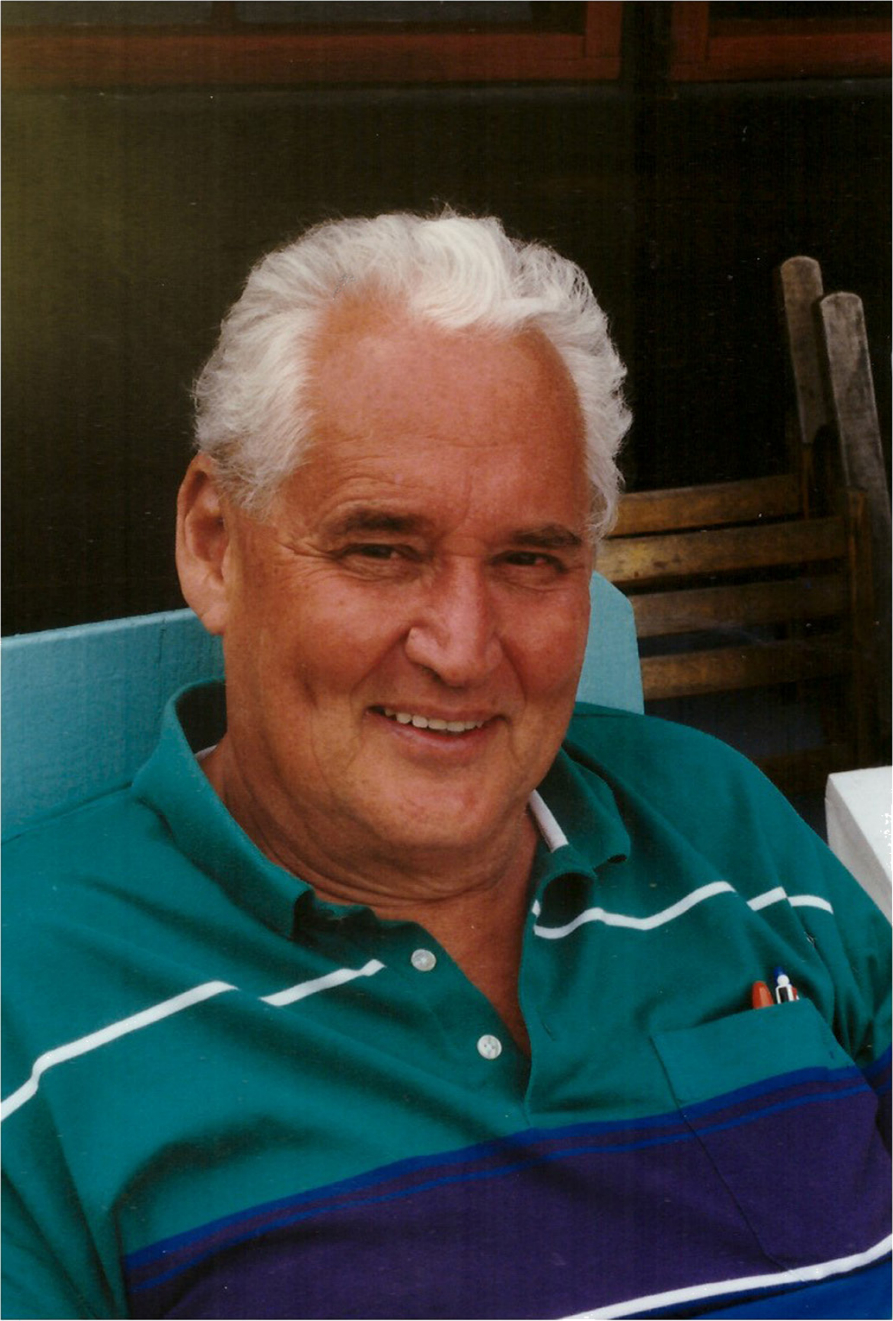


Prof. Jacques-Emile Dumont. Photograph courtesy Jacqueline Dumont.

Jacques-Emile Dumont passed away on February 6, at the age of 91. He was Honorary Professor of Biochemistry and Endocrinology at the Faculty of Medicine and the Faculty of Sciences of the Free University Brussels (ULB), and had been President of the ETA between 1996 and 1998.

At the end of the 1950s after a postdoctoral stay in John Stanbury’s laboratory in Boston, JED (as he would usually sign) came back to the Free University Brussels and joined the laboratory of experimental medicine of PP Bastenie, one of the initiators of thyroid research in Belgium. With the novel availability of radioiodine, he co-founded with André Ermans the Laboratory of Nuclear Medicine (LMN) in 1963. André Ermans, himself very active in thyroid research, would go on using radioisotopes in the hospital, while JED would orient the LMN to basic research, keeping the strong links initially established with a series of clinicians. Over the years, he transformed the LMN into what became the Institute of Interdisciplinary Research in Human and Molecular Biology (IRIBHM), the main research institute of the Faculty of Medicine of ULB, which he directed from 1968 to 2001. In the 1970s, the Institute attracted people from Japan, the USA, Canada and South America to study problems related to the thyroid. Today, its PhDs, postdocs and PIs constitute a busy Tower of Babel where researchers from 14 countries work together on a wide spectrum of subjects.

Jacques Dumont’s first contribution to thyroid research related to the pathophysiology of myxoedematous cretinism, which he studied with François Delange and André Ermans during several perilous trips to Zaïre, in the tumult of the decolonization of the Belgian Congo. He continued to pursue this subject and decades later discovered the role of selenium in the pathogenesis of endemic cretinism. Thereafter, his main scientific achievements were the result of studies performed under his guidance in the Institute he created and where he consistently implemented the most up-to-date technology. Amongst the more important are as follows: dissection of the signal transduction pathways and the role of cyclic AMP in the thyrocyte; identification of iodinated intermediates involved in the negative regulation of thyroid function; demonstration of a mitogenic action of cyclic AMP on the thyrocyte; cloning of the main thyroid autoantigens and identification of gain- and loss-of-function mutations responsible for genetic causes of hyper- and hypothyroidism; identification and cloning of the enzyme involved in H_2_O_2_ generation; and generation of several transgenic mouse models of thyroid cancer. Despite official retirement (compulsory in Belgium), JED continued until recently to provide guidance on his favorite research dealing with thyroid cell proliferation, cancer and thyroid physiology.

Jacques Dumont was literally an extraordinary character with many different facets. One of his main traits was certainly his drive ‘to build’: as such, he was amongst the founders of the ETA and he created his research institute, first in old interconnected private houses downtown Brussels and then in the first faculty building of a new ULB campus; in the 1990s, he co-founded two successful spinoffs, EUROSCREEN and CHEMCOM, which exploited results unrelated to thyroid research produced in his Institute and contributed significantly to research funding; in 1977, he co-founded the Hormone and Cell Regulation symposium, which still gathers each year in Mont Ste Odile (France). Another trait was his competitive mind, which derives probably from his activity as a basketball player (which he played well into in his sixties). He also displayed a strong aptitude for indignation to injustice, inequality and stupidity and, contrary to many, his indignation increased with age. JED was a born leader, capable of inspiring the most placid personalities. His capacity to support and motivate people was paramount especially when, as is routine in research, things do not turn out as expected. For over 40 years, and beyond the limits of his Institute, he was the leader of an informal group of individuals who still today recognize and share his values.

His combative and lively temperament made for many good days at meetings. We will miss him, standing up at scientific sessions or general assemblies, with questions or remarks that shook the audience.

Our thoughts go to Jacqueline, his wife and closest collaborator.

On behalf of:

*all present and past members of the IRIBHM*

his friend *Gilbert Vassart*

